# Identification of transcriptome signature for predicting clinical response to bevacizumab in recurrent glioblastoma

**DOI:** 10.1002/cam4.1439

**Published:** 2018-03-23

**Authors:** Seung Won Choi, Hyemi Shin, Jason K. Sa, Hee Jin Cho, Harim Koo, Doo‐Sik Kong, Ho Jun Seol, Do‐Hyun Nam

**Affiliations:** ^1^ Department of Health Sciences and Technology SAIHST Sungkyunkwan University Seoul Korea; ^2^ Institute for Refractory Cancer Research Samsung Medical Center Seoul Korea; ^3^ Department of Neurosurgery Samsung Medical Center Sungkyunkwan University School of Medicine Seoul Korea

**Keywords:** Angiogenesis, bevacizumab, biomarkers, gene expression signatures, glioblastoma

## Abstract

Glioblastomas are among the most fatal brain tumors. Although no effective treatment option is available for recurrent glioblastomas (GBMs), a subset of patients evidently derived clinical benefit from bevacizumab, a monoclonal antibody against vascular endothelial growth factor. We retrospectively reviewed patients with recurrent GBM who received bevacizumab to identify biomarkers for predicting clinical response to bevacizumab. Following defined criteria, the patients were categorized into two clinical response groups, and their genetic and transcriptomic results were compared. Angiogenesis‐related gene sets were upregulated in both responders and nonresponders, whereas genes for each corresponding angiogenesis pathway were distinct from one another. Two gene sets were made, namely, the nonresponder angiogenesis gene set (NAG) and responder angiogenesis gene set (RAG), and then implemented in independent GBM cohort to validate our dataset. A similar association between the corresponding gene set and survival was observed. In NAG, COL4A2 was associated with a poor clinical outcome in bevacizumab‐treated patients. This study demonstrates that angiogenesis‐associated gene sets are composed of distinct subsets with diverse biological roles and they represent different clinical responses to anti‐angiogenic therapy. Enrichment of a distinct angiogenesis pathway may serve as a biomarker to predict patients who will derive a clinical benefit from bevacizumab.

## Introduction

Glioblastomas (GBMs) are one of the most malignant and fatal brain cancers, with a median survival time of only 12–15 months 1. Despite intensive standard care, almost all patients with GBM eventually experience tumor relapse, and no effective treatment strategy against recurrent GBM tumors has been established; thus, a new therapeutic approach is needed. Bevacizumab, a monoclonal antibody against vascular endothelial growth factor (VEGF), has been anticipated to provide prognostic benefits in patients with GBM. However, several large clinical trials have failed to demonstrate the clinical benefit of bevacizumab in terms of overall survival (OS) 2, 3, 4, 5, 6. Although the number of responders is limited, a subset of patients with GBM evidently demonstrated clinical benefits from bevacizumab therapy. Thus, several studies aimed to identify new biomarkers for predicting clinical response to bevacizumab have been conducted. However, robust predictive markers are yet to be identified. In this study, we retrospectively reviewed patients with GBM who received bevacizumab during recurrence to determine the genomic traits that present targeted vulnerability to bevacizumab.

## Materials and Methods

### Patient‐derived GBM specimens

This study was approved by the institutional review board of Samsung Medical Center, and written informed consent was obtained from all patients. Surgical specimens and clinical information were obtained from patients with GBM who underwent brain tumor surgery at Samsung Medical Center. For genomic analysis, sections of the tumor specimens were snap‐frozen and preserved in liquid nitrogen until use. Genomic DNA and mRNA were extracted using the DNeasy kit and the RNeasy kit (Qiagen, Hilden, Germany), respectively. Patients who were treated with at least one course of bevacizumab for their recurrent GBM were included, whereas those without RNA‐seq data or any available follow‐up magnetic resonance imaging (MRI) to assess the postbevacizumab response were excluded. Tumor samples analyzed for genomic alteration, including whole‐exome sequencing, target‐exome sequencing, and RNA sequencing, in this study were obtained from the initial diagnostic surgery of newly diagnosed patients. Recurrent samples are the best candidate to represent the genetic background of sensitivity to bevacizumab in the recurrent setting of glioblastoma. However, because of the retrospective nature of this study, we could not collect corresponding recurrent samples for RNA sequencing. Patients were categorized into two groups according to clinical response to bevacizumab, and the criteria for differentiating the clinical response among patients are detailed below.

### Clinical response

In clinical practice, tumor progression is usually defined by both clinical deterioration and MR images. However, it remains challenging to distinguish radiation necrosis from true progression via MR images alone, and clinical symptoms cannot be used as a robust indicator to differentiate true progression from radiation necrosis. Patients with radiation necrosis also experience clinical deterioration; however, such symptoms usually resolve within a short period in contrast to true tumor progression. Bevacizumab is effective in controlling edema accompanied by radiation necrosis 7, and such cases of radiation necrosis treated by bevacizumab can be a significant confounder to this study.

To address such challenges, we combined two methodological criteria, namely MR response by RANO criteria and progression‐free survival (PFS), to categorize patients into two clinical response groups: responders and nonresponders to bevacizumab. Only few cases had relatively long PFS, and these cases may represent radiation necrosis. All patients in this group maintained clinical benefits for more than 3 months after discontinuing bevacizumab, strongly suggesting that these cases were more likely to be radiation necrosis rather than true tumor progression.

### RNA sequencing

RNA sequencing libraries were prepared using the Illumina TruSeq RNA Library Preparation Kit v2. The sequenced reads were trimmed and mapped onto hg19 using GSNAP version 2012‐12‐20 8. The resulting aligned reads were summarized into BED files using SAMtools and bedTools (bamToBed version 2.16.2) 9. The BED files were used to estimate reads per kilobase of transcript per million reads (RPKM) using the R package DEGseq 10.

DEGseq: Differentially expressed genes were identified using the R package “DEGseq” 10. Samples were divided into two groups according to clinical response to bevacizumab.
ssGSEA: Single‐sample gene set enrichment analysis (ssGSEA) was used to estimate the enrichment score of each sample using GenePattern software of Broad Institute (http://software.broadinstitute.org/cancer/software/genepattern). Gene expression data were normalized using a “rank” method 11. The subtype of each sample was assigned using z‐score 1.

### Whole‐exome sequencing and target‐exome sequencing

#### Somatic mutation

The sequenced reads in the FASTQ files were aligned to the human genome assembly (hg19) using Burrows–Wheeler aligner. The initial alignment BAM files were preprocessed for sorting, removing duplicate reads, realigning reads around potential small indels, and recalibrating base quality score using SAMtools. MuTect and Somatic IndelDetector were used to make high‐confidence predictions on somatic mutations from neoplastic and non‐neoplastic tissue pairs. Variant effector predictor was used to annotate somatic mutations.

### Statistical analysis

All values are shown as the mean ± standard deviation. Continuous variables were compared using Wilcox rank‐sum test, and categorical variables were tested using Fisher's exact test. Survival analysis was performed using a Kaplan–Meier plot, and survival curves were statistically tested using the log‐rank test. All statistical analyses were performed using R software version 3.4.2 (http://www.R-project.org) (Team, R.C.R: A language and environment for statistical computing. R foundation for statistical computing, Vienna, Austria. 2013 (2014)). *P* < 0.05 was considered to indicate a statistically significant difference.

## Result

### Patients’ profile

According to the defined response criteria, nine patients were categorized into either responder (*n* = 5) or nonresponder group (*n* = 4). The clinical features of each patient are summarized in Table 1. The median age at initial diagnosis was 58 years (range, 42–72 years), and majority of the patients (6 of 9, 66.7%) were treated with bevacizumab for their second GBM recurrence. The median PFS of the nonresponder and responder groups was 33 and 143 days, respectively (log‐rank test, *P* = 0.0027). Among patients in this study, G606 patient demonstrated remarkable response to bevacizumab, has shown a sustained partial response for more than 10 months, and continues to show clinical benefit until now. This patient is still on bevacizumab therapy currently.

**Table 1:** Clinical features of responder versus nonresponder group

Patient	Sex/age	Prior therapy	When to start Bevacizumab	No. of BEZ treatment	BEZ response
N520	M/42	CCRT+#4 + surgery	On 2nd recur	3	Nonresponder
G352	F/63	CCRT+LDTMZ#4 + afatinib	On 2nd recur	2	Nonresponder
G074	M/60	CCRT+#6 + GKRS+LDTMZ#4	On 2nd recur	2	Nonresponder
G287	F/72	CCRT+#6 + RT	On 2nd recur	2	Nonresponder
B870	M/43	CCRT+#4	On 1st recur	15	Responder
G606	F/58	CCRT+#6 + op+CCRT+#2 + op + RT + GKRS+RT	On 2nd recur	14^a^	Responder
G364	F/60	CCRT+#6	On 1st recur	18	Responder
N538	F/42	CCRT+#6 + surgery + LDTMZ#6 + GKRS	On 3rd recur	10	Responder
G406	F/53	CCRT+#3 + LDTMZ#4	On 2nd recur	10	Responder

BEZ, bevacizumab; CCRT, concurrent chemoradiotherapy; GKRS, gamma knife radiosurgery; LDTMZ, low‐dose temozolomide; RT, radiation therapy.

a Marked represents a censored data, indicating that G606 patient is still on the BEZ with partial response currently.

**Figure 1:**
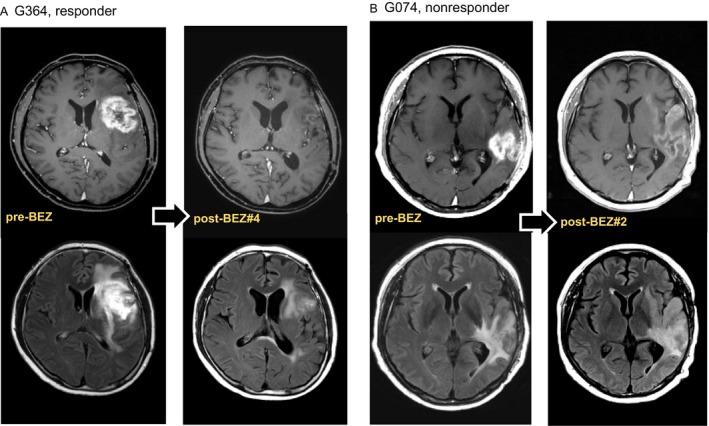
MRIs following BEZ treatment. (A) G364 demonstrated remarkable decrease in contrast enhancement and T2 infiltration accompanied with edema following bevacizumab, while both contrast enhancement and T2 infiltration of G074 (B) had been maintained and somewhat increased despite bevacizumab. MRI, magnetic resonance image; BEZ, bevacizumab; pre‐BEZ, before treatment of bevacizumab; post‐BEZ#2, after 2 cycles of bevacizumab treatment.

Patients in the responder group exhibited substantial responses to bevacizumab, whereas those in the nonresponder group showed rather constant disease progression (Fig. 1). In the responder group, the contrast‐enhanced lesion as well as the T2 infiltrative lesion showed a remarkable decrease in response to bevacizumab. Meanwhile, despite bevacizumab therapy, both contrast enhancement and T2 FLAIR lesions have increased and demonstrated more invasive and infiltrative radiologic features on follow‐up MRI in the nonresponder group. No patient in our series demonstrated a “pseudoresponse” 12, that is, increased T2 FLAIR infiltration, whereas a decrease in contrast‐enhanced lesions.

### Genomic landscape of responder versus nonresponder

We analyzed the somatic mutations and copy number alterations in the major oncogenic pathways of GBM 13 in both responder and nonresponder groups. Genomic profiling did not demonstrate any distinguishable somatic variants between the two groups (Table 2). However, transcriptome analysis of glioma‐intrinsic gene expression subtypes revealed a notable distinction between the two groups 14. Majority of the nonresponder tumors were classified as the classical subtype (3 of 4, 75%), whereas responder tumors were mainly nonclassical (1 of 5, 20%, *P* = 0.333). Although statistically not significant, our results were consistent with previous studies, demonstrating a correlation between clinical resistance to bevacizumab and classical subtype 15.

**Table 2:**
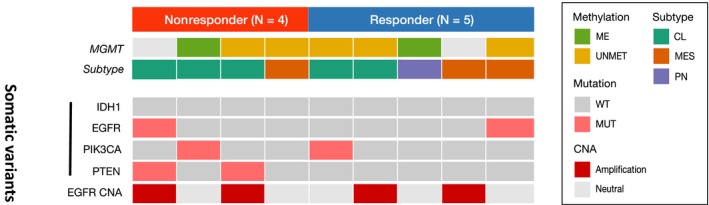
Genomic landscape of responder and nonresponder

CL, classical; CNA, copy number alteration; ME, methylated; MES, mesenchymal; MUT, mutation; PN, proneural; UNMET, unmethylated; WT, wild‐type.

### Enrichment of angiogenesis‐related gene sets

**Figure 2:**
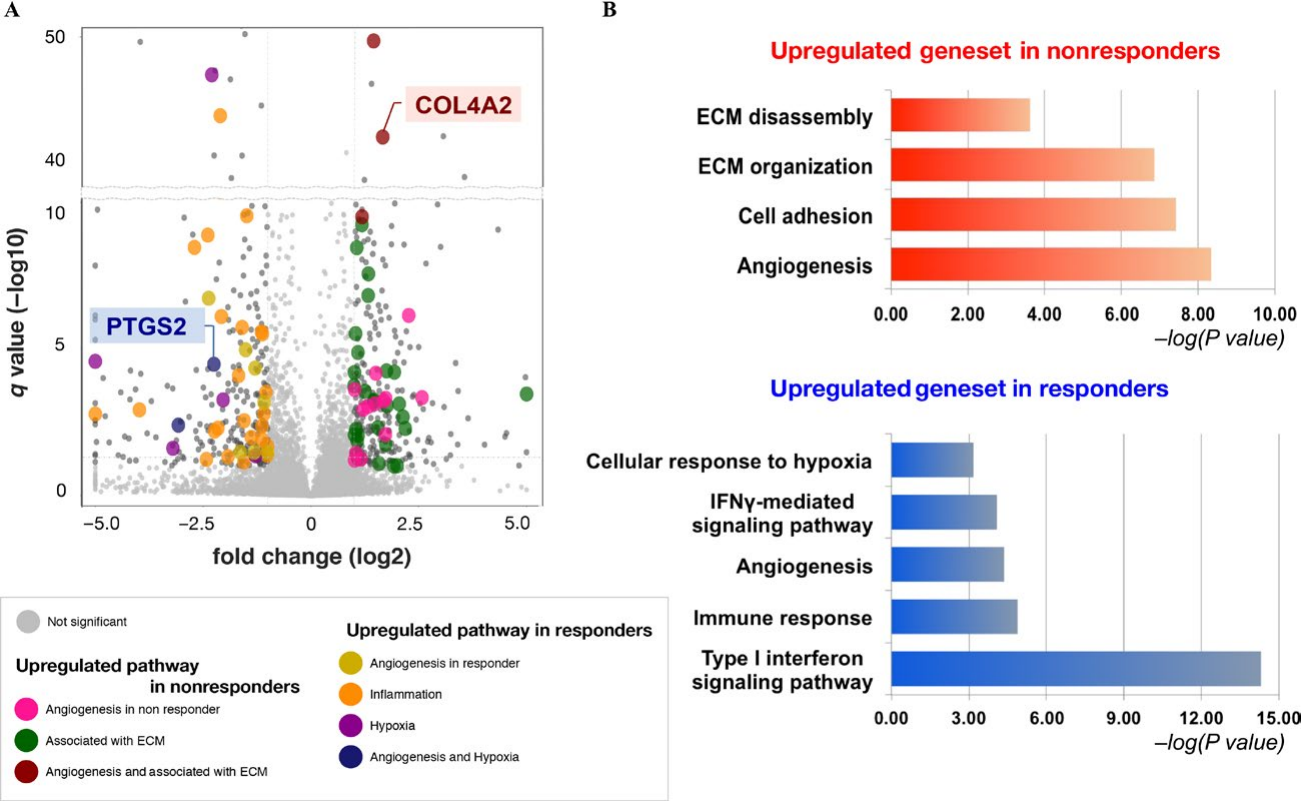
Upregulated genes and associated GO pathways in responder versus nonresponder group. (A) Differentially expressed genes between responder and nonresponder groups are plotted as a volcano plot. Genes within the same genetic pathways are represented using the same color scale. Dots in the right side of the graph depict genes that are upregulated in nonresponders while dots in the left side demonstrate genes upregulated in responders. (B) Upregulated genes in each group are functionally annotated with GO terminology, and top pathways with statistical significance are illustrated. ECM, extracellular matrix.

To identify the extent of differential transcriptomic traits that drive responding and nonresponding tumors, the corresponding nine tumor specimens were subjected for whole‐transcriptome sequencing. Transcriptome analysis revealed a set of significantly differentially expressed genes, including PTGS2 and COL4A2 (Fig. 2A). Functional annotation of differentially expressed genes between the two groups using GO terminology identified potential underlying mechanisms that may direct clinical response of bevacizumab. Type 1 interferon pathway, immune response, and angiogenesis were significantly upregulated in the responder group (*P* = 4.90E−15, 1.30E−5, and 4.30E−5, respectively), while angiogenesis and extracellular matrix disassembly were upregulated in the nonresponder group (*P* = 4.60E−09 and 6.10E−05, respectively). Interestingly, angiogenesis‐associated gene sets were upregulated in both groups, suggesting diverse functional roles of angiogenesis. Genes comprising each corresponding angiogenesis pathway were distinct from each other. Our result seemed unconventional at first as activation of proangiogenic pathway is often suggested as one of the underlying mechanisms of acquiring bevacizumab resistance 16.

**Figure 3:**
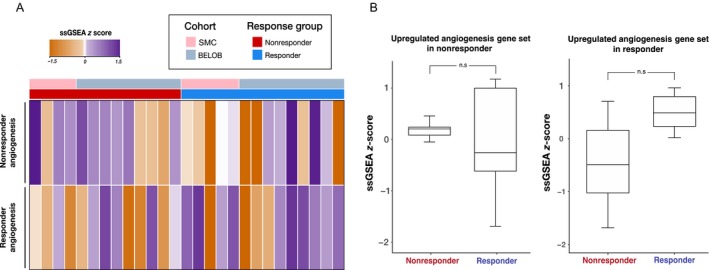
Responders and nonresponders have distinct angiogenesis‐related gene sets. Two angiogenesis‐associated gene sets regarding the clinical prognosis to bevacizumab therapy are made and termed as responder angiogenesis gene set (RAGs) and nonresponder angiogenesis gene set (NAGs). (A) We applied these gene sets to the independent glioblastoma cohort from the BELOB trial. The ssGSEA scores of each gene set are represented as Z‐scores. (B) RAGs expression was upregulated in responders (long‐term survivors), whereas NAGs expression was upregulated in nonresponders (short‐term survivors) of the BELOB trial. Short‐ and long‐term survivors are defined as follows: short‐term survivors denote patients who survived for less than 25% of the survival period of the entire cohort. Long‐term survivors are patients who survived for more than 75% of the overall survival of entire cohort. Error bars mean standard deviation of ssGSEA Z‐scores and n.s refer statistical insignificance from Wilcoxon test. ssGSEA, single‐sample gene set enrichment analysis; RAG, responder angiogenesis gene set; NAG, nonresponder angiogenesis gene set.

After bevacizumab therapy, patients with GBM frequently present with a more invasive and infiltrative disease than that before therapy 17, 18, 19, indicating that bevacizumab may induce a malignant phenotypic state. Such a discrepancy is consistently observed in other tumors as well, suggesting potential bevacizumab addiction of the tumor growth and invasion 20, 21, that is, tumors seem to be dependent on bevacizumab for their growth and invasion following bevacizumab therapy. The generalized underlying mechanism of anti‐angiogenic therapy involves vessel normalization 22. Malignant phenotypic transformation following anti‐angiogenic therapy resembles hypoxia‐induced state that has little relevance to vessel normalization 23. Thus, we suspected that two functionally different angiogenesis pathways may co‐exist; one of them is involved in vessel normalization, whereas the other regulates tumorigenic angiogenesis. Therefore, differential expression of these pathways may cause diverse clinical response to anti‐angiogenic therapy. To validate our findings, we applied our acquired angiogenesis‐associated gene sets, which we termed responder angiogenesis gene set (RAGs) and nonresponder angiogenesis gene set (NAGs), to the independent GBM cohort. In the BELOB trial 24, patients with recurrent GBM were randomly assigned to three treatment arms: lomustine (CCNU), bevacizumab, or a combination of CCNU and bevacizumab 25. Patients who were assigned to bevacizumab single therapy were selected and categorized according to OS. Notably, RAGs expression was upregulated in long‐term survivors, that is, those who survived for more than 75% of the OS of the entire cohort. Meanwhile, NAGs expression was upregulated in short‐term survivors, that is, those who survived for only 25% of the survival period of the entire cohort. These results further corroborate our findings (Fig. 3).

### COL4A2 is associated with poor clinical outcome in patients who receive bevacizumab treatment

**Figure 4:**
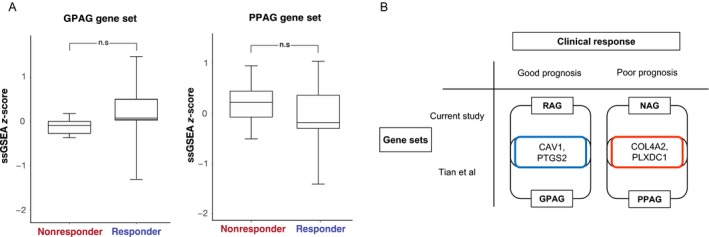
Good‐prognosis angiogenesis genes (GPAGs) and poor‐prognosis angiogenesis genes (PPAGs) scores in the SMC glioblastoma cohort. Tian et al. identified two distinct subsets related to clinical prognosis within the angiogenesis‐associated genes and defined them as GPAG and PPAG. These two gene sets are applied to our cohort 26. (A) GPAGs and PPAGs are implemented to our glioblastoma cohort and showed similar association between prognosis and corresponding gene sets. (B) A few genes are shared between GPAG/PPAG and NAG/RAGs. SMC, Samsung Medical Center; ssGSEA, single‐sample gene set enrichment analysis; GPAG, good‐prognosis angiogenesis genes; PPAG, poor‐prognosis angiogenesis genes; RAG, responder angiogenesis gene set; NAG, nonresponder angiogenesis gene set.

**Figure 5:**
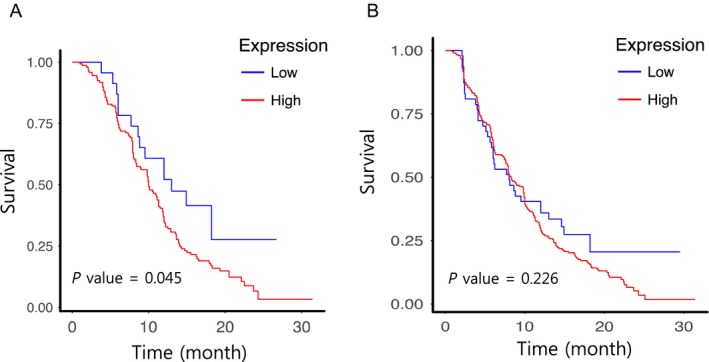
COL4A2 is associated with poor prognosis of glioblastoma with BEZ treatment. Public data from AVAglio+RTOG0825 (Gene Expression Omnibus; Access number: GSE84010 (https://www.ncbi.nlm.nih.gov/geo)) are used to validate the prognostic significance of COL4A2 in BEZ‐treated patients with GBM. (A) COL4A2 expression and overall survival of the patients with GBM treated with BEZ. Patients treated with BEZ are stratified according to COL4A2 mRNA expression. (B) COL4A2 expression and overall survival of the total glioblastoma dataset regardless of BEZ treatment. Patients from the entire cohort stratified according to COL4A2 mRNA expression. BEZ, bevacizumab; GBM, glioblastoma.

Two distinct angiogenic‐associated gene sets, RAGs and NAGs, suggest potential opposing roles of angiogenesis in promoting tumor vascularization, underscoring the importance of comprehending two different clinical outcomes of malignancy following anti‐angiogenic therapy. Notably, a recent study 26 has identified two distinct subsets of angiogenesis genes that are associated with either favorable or dismal prognosis in breast cancer, namely, good‐prognosis angiogenesis genes (GPAGs) and poor‐prognosis angiogenesis genes (PPAGs), respectively. GPAGs reflect vessel normalization, whereas PPAGs are mostly related to protumoral events such as extracellular matrix disassembly and hypoxia. Such observations were consistent with our findings; therefore, we applied these gene sets to our cohort to evaluate whether the results were coherent. Although it was not statistically significant, GPAGs were upregulated in the responder group, whereas PPAGs were upregulated in the nonresponder group, which was consistent with our hypothesis (Fig. 4). Accordingly, we further suspected that our two generated gene sets, RAGs and NAGs, could share common grounds with GPAGs and PPAGs as both gene sets could predict the prognosis of patients with GBM in response to bevacizumab treatment. Interestingly, we have identified several genes that were present in both independent gene sets, namely, *CAV1*,* PTGS2*,* PLXDC1*, and *COL4A2*. To determine whether the resulting genes could serve as prognostic markers for patient survival, we stratified patients with GBM from an independent cohort (AVAglio + RTOG 0825) according to the transcriptome expression of the corresponding gene 25, 27. Notably, *COL4A2* mRNA expression was significantly correlated with the OS of the patients who received bevacizumab treatment (Fig. 5). Additionally, the prognostic significance of *COL4A2* expression was absent when applied to the entire cohort regardless of bevacizumab therapy, suggesting *COL4A2* as a bevacizumab‐specific predictive marker.

## Discussion

GBM is one of the most lethal brain tumors with dismal prognosis. Majority of the patients with GBM eventually develop tumor recurrence, and no effective therapeutic strategy has been determined to date. Prominent histopathological and genomic features of GBM include rapid vascularization, infiltrative growth, and aberrant activation of VEGF‐A 28, 29, 30. Therefore, bevacizumab, a monoclonal antibody to VEGF‐A, has been anticipated as a potent and selective agent against GBM progression. However, numerous clinical trials have failed to demonstrate any clinical benefit of bevacizumab in both newly diagnosed and recurrent GBM. Only a subset of patients showed favorable clinical response following bevacizumab, and numerous studies aimed to identify new biomarkers for predicting clinical response to bevacizumab have been conducted.

We conducted a retrospective study on patients who received bevacizumab treatment to identify genomic and transcriptomic traits that present targeted vulnerability to bevacizumab. Clinical response following bevacizumab was determined using radiologic response and PFS. Defining a robust and strict outline to distinguish between bevacizumab response and nonresponse in real clinical practice is challenging as most recurrent tumors are treated only on the basis of clinical guidelines, without confirmative histologic evidence of recurrent tumor. Among the patients who were treated with bevacizumab based on recognition of recurrent GBM, some patients have shown an extremely long‐term clinical response (Fig. S1). All these patients maintained clinical benefit for more than 3 months after stopping bevacizumab and were thus considered to harbor a radiation necrosis rather than recurrent tumor. Although advanced MRI techniques may provide supplementary information for the differential diagnosis of true progression from tumor recurrence, it still remains extremely challenging to accurately differentiate radiation necrosis from true tumor progression. As such, we employed strict clinical criteria using both PFS and radiologic response to select genuine representative responder and nonresponder cases.

Genomic profiling of corresponding tumors did not demonstrate any significant difference between responders and nonresponders in terms of core oncogenic pathways that are frequently altered in GBM. However, transcriptome analysis showed a difference in terms of molecular subtype consistent with previous studies 15, majority of the nonresponders were classified as classical subtype, while responder cases were mainly comprised of the nonclassical subtype. Notably, two large independent clinical trials have reported the predictive role of transcriptional molecular subtype in clinical response 25, 27. Erdem‐Erason et al. discovered that classical subtype recurrent GBMs showed significant clinical benefits to bevacizumab treatment in terms of PFS. Furthermore, Sandman et al. reported that bevacizumab conferred a significant OS advantage in patients with proneural *IDH1* wild‐type GBM. However, the above studies were conducted in different settings of disease presentation and treatment strategy; combination treatment including bevacizumab and CCNU was given for recurrent GBM in the BELOB trial, while the AVAglio trial evaluated the addition of bevacizumab versus placebo to standard treatment (radiotherapy with concurrent temozolomide) for newly diagnosed GBM. Thus, discordant result from these two trials was expected and cannot be directly compared to our current study that consists of bevacizumab monotherapy for recurrent tumors. However, important lessons are to be learned as phenotypic expression such as molecular subtype may represent different clinical response following bevacizumab. Laffaire et al. reported that recurrent GBM assigned to IGS‐18 or classical subtype showed unfavorable clinical response to bevacizumab, independent of *EGFR* amplification or *CDKN2A* deletion 15. Although statistically insignificant, a similar trend of association between clinical resistance and classical subtype was also seen in our present study. However, this result should be interpreted with caution as molecular subtype at initial presentation may be altered upon recurrence.

Vessel normalization is proposed as the primary mechanism of action behind anti‐angiogenic therapy including bevacizumab 22, and majority of the patients with GBM exhibit decreased contrast enhancement following bevacizumab. However, upon recurrence, most tumors undergo malignant transformation, displaying more aggressive phenotypic state. T2 infiltration often precedes overwhelming contrast enhancement and shows features similar to those of the hypoxia‐induced state 23. However, the underlying mechanism of post‐anti‐angiogenic hypoxia has been debated as it is often related to vessel normalization, which is contradictory to previous observations.

In the present study, transcriptome analysis between two clinical groups revealed two distinct angiogenesis‐associated gene sets that were upregulated in both groups. Upregulation of angiogenic signature in nonresponders could be interpreted as activation of the proangiogenic pathway, which is often responsible for resistance to anti‐angiogenic therapy 16. However, alternative angiogenic signature, upregulated in responders, is contrary to previous findings. Based on clinical and transcriptome discrepancy, we postulated that angiogenesis‐related genes were composed of distinct subgroups that potentially possess diverse functional roles in promoting vessel normalization. A recent study has focused on mutual interaction between type 1 T helper cell and angiogenesis pathway that resulted in identification of two distinct gene sets, in which both of them were associated with angiogenesis, consistent with our findings. To test our hypothesis, we applied our generated angiogenic gene sets (RAG and NAG) to the independent cohort of BELOB trial. Although statistically insignificant, RAG was highly upregulated in long‐term survivors, whereas NAG was more upregulated in short‐term survivors.

Tian et al. 26 previously established two angiogenesis‐associated gene sets that have distinct biological and functional roles in vessel normalization and prognosis. These gene sets were termed as GPAG and PPAG. When we implemented these two gene sets to our current cohort, we observed a consistent result in terms of clinical prognosis. Notably, we have identified *COL4A2* as one of the overlapped gene between our generated gene sets and GPAG/PPAG, and it demonstrated significant prognostic value in patients treated with bevacizumab.

*COL4A2* encodes for alpha‐2 chain of type IV collagen, which is a major component of basement membrane. Previous studies have demonstrated the pivotal role of *COL4A2* in vascular stability 31, affecting intracerebral hemorrhage 32, 33, 34. In the present study, high *COL4A2* mRNA expression significantly correlated with dismal prognosis in bevacizumab‐treated patients, which can be attributed to the essential role of *COL4A2* in maintaining vascular stability.

To confirm whether the transcriptomic traits we used to represent the clinical response to bevacizumab is maintained through the tumor evolution, we analyzed the separate set of longitudinal GBM pair samples in our institute. Each pair is composed of primary and recurrent samples from one patient. We calculated the single‐sample GSEA scores of RAG and NAG of 29 longitudinal pairs. Each score was normalized into Z‐score, and we assigned each sample into responder or nonresponder according to its Z‐transformed ssGSEA score. Majority of patients (79%, 23 of 29) maintained their transcriptomic traits in terms of sensitivity to bevacizumab. Moreover, we also compared the gene expression of COL4A2 and PTGS2 in the same longitudinal pairs and found no significant difference in the expression of these two genes between the primary and recurrent tumors (*P* = 0.152 and 0.062 for COL4A2 and PTGS2, respectively, paired *t*‐test). These findings support that the transcriptomic traits from the primary tumors that we used to portray the clinical response to bevacizumab may replace that of recurrent tumors.

The current study has some limitations, including its retrospective nature and small number of cases. Tumor samples used in this study were acquired at initial presentation of each corresponding patient. Tumor evolution during therapy is another major concern in evaluating the sensitivity to further treatment 35, 36. The genetic landscape of tumors may be altered along with tumor evolution 35, 36. In particular, the molecular subtype, which is determined by transcriptomic expression, is well known to be altered during recurrence in GBM 36, 37. Therefore, tumor samples should be obtained at an optimal timing to accurately determine the genetic trait for clinical response to bevacizumab during recurrence. Because of the retrospective nature of this study, we could not collect enough corresponding recurrent samples for genomic analysis. Moreover, tumor evolution from initial diagnosis until recurrence can be a confounding factor in this study. In clinical practice, redo surgery is usually performed in patients with more favorable prognostic factors, while bevacizumab is usually reserved for salvage therapy rather than radical purposes. Obtaining immediate recurrent tumor samples before bevacizumab therapy is not usually feasible in real clinical practice.

However, despite these limitations, one of the major findings in this study is that angiogenesis‐associated gene sets are composed of distinct subsets with diverse biological roles. Furthermore, each subset of angiogenesis‐associated genes can portray different clinical response to anti‐angiogenic therapy. Such a concept of dual functional roles in angiogenesis‐associated genes may help investigate the potential underlying mechanisms of varying clinical responses following anti‐angiogenic therapy.

## Conflict of Interest

The authors have no potential conflict of interests to disclose.

## Supporting information


**Figure S1.** Tumor samples were selected by clinical response (PFS) and radiologic response following BEZ.
