# Supplementary material for: Identification of transcriptome signature for predicting clinical response to bevacizumab in recurrent glioblastoma
Source: Cancer Med. 2018 Mar 23;7(5):1774–83. doi: 10.1002/cam4.1439 (PMC5943425; doi:10.1002/cam4.1439)
Supplement: Supplementary file 1 — Figure S1. Tumor samples were selected by clinical response (PFS) and radiologic response following BEZ. [file CAM4-7-1774-s001.pdf]

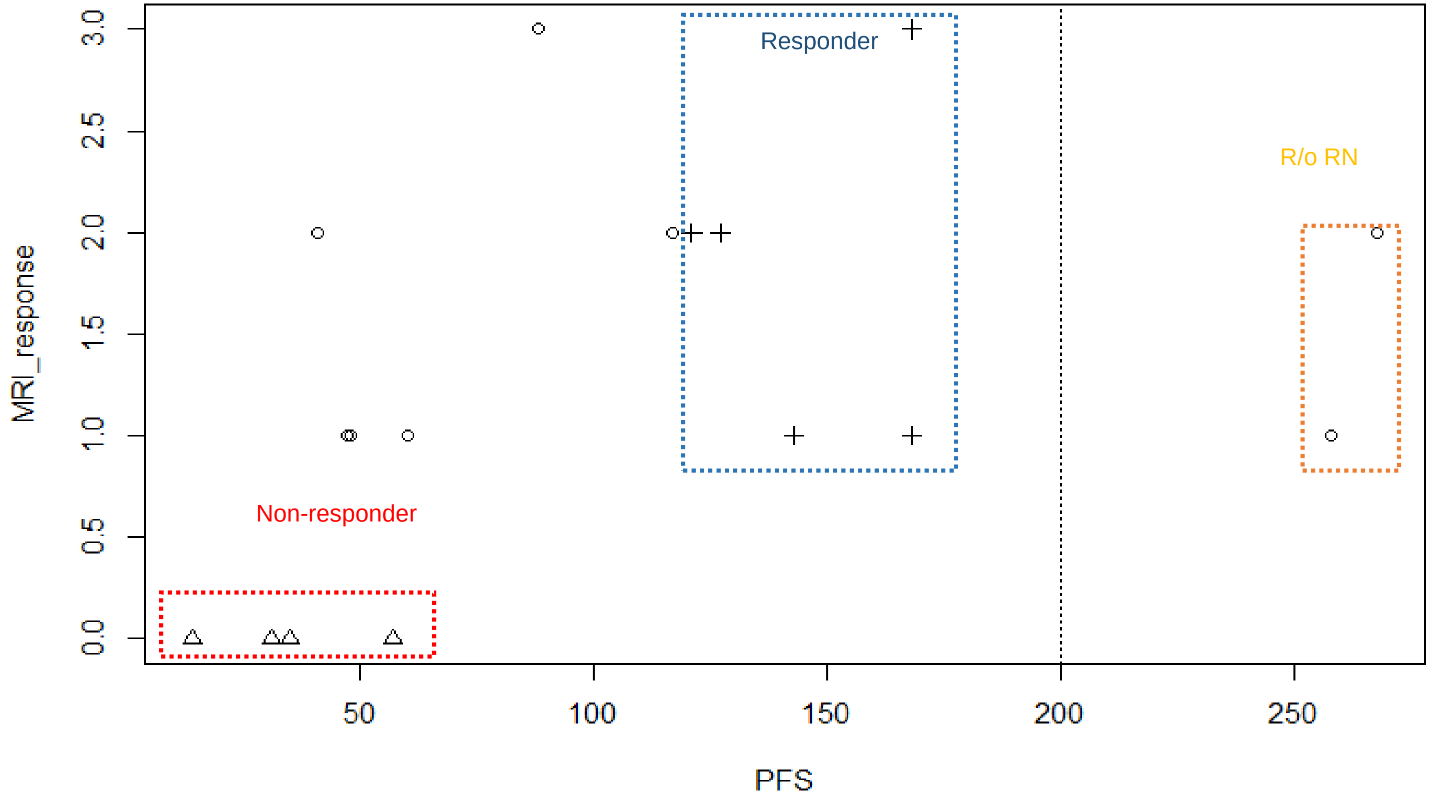

Supplementary Figure 1. Tumor samples were selected by clinical response (PFS) and radiologic response following BEZ.

Abbreviation : RN, radiation necrosis; PFS, progression free survival(days); BEZ, bevacizumab
